# Next-Generation Sequencing Highlights of Diffuse Large B-cell Lymphoma in a Tertiary Care Hospital in North India

**DOI:** 10.7759/cureus.28241

**Published:** 2022-08-21

**Authors:** Garima Mamgain, Manisha Naithani, Priyanka Patra, Mukesh Mamgain, Sikha Morang, Jhasketan Nayak, Karthik Kumar, Shashikant Singh, Anamika Bakliwal, Ashok Rajoreya, Sudeep Vaniyath, Debranjani Chattopadhyay, Rituparna Chetia, Arvind Gupta, Gaurav Dhingra, Deepak Sundriyal, Uttam Kumar Nath

**Affiliations:** 1 Department of Medical Oncology/Haematology, All India Institute of Medical Sciences, Rishikesh, Rishikesh, IND; 2 Department of Biochemistry, All India Institute of Medical Sciences, Rishikesh, Rishikesh, IND; 3 Department of Pharmacology, All India Institute of Medical Sciences, Rishikesh, Rishikesh, IND; 4 Department of Pathology, All India Institute of Medical Sciences, Rishikesh, Rishikesh, IND

**Keywords:** informativity, lymphoma panel, genetic mutation, next-generation sequencing, diffuse large b cell lymphoma

## Abstract

Introduction: Next-generation sequencing (NGS) elucidates the diffuse large B-cell lymphoma (DLBCL) genetic characteristics by finding recurrent and novel somatic mutations. This observational study attempted to create an NGS panel with a focus on identifying novel somatic mutations which could have potential clinical and therapeutic implications. This panel was created to look for mutations in 133 genes chosen on basis of a literature review and it was used to sequence the tumor DNA of 20 DLBCL patients after a centralized histopathologic review.

Methods: The study included 20 patients having DLBCL. The quality and quantity of tumor cells were accessed by H&E staining and correlated with histopathology and Immunohistochemistry (IHC) status. Patients were grouped as ABC (activated B-cell), PMBL (primary mediastinal large B-cell lymphoma), and other or unclassified subtypes. The lymphoma panel of 133 was designed on targeted sequencing of multiple genes for the coding regions through NGS. The libraries were prepared and sequenced using the Illumina platform. The alignment of obtained sequences was performed using Burrows-Wheeler Aligner and identification of somatic mutations was done using LoFreq (version 2) variant caller. The mutations were annotated using an annotation pipeline (VariMAT). Previously published literature and databases were used for the annotation of clinically relevant mutations. The common variants were filtered for reporting based on the presence in various population databases (1000G, ExAC, EVS, 1000Japanese, dbSNP, UK10K, MedVarDb). A custom read-depth-based algorithm was used to determine CNV (Copy Number Variants) from targeted sequencing experiments. Rare CNVs were detected using a comparison of the test data read-depths with the matched reference dataset. Reportable mutations were prioritized and prepared based on AMP-ASCO-CAP (Association for Molecular Pathology-American Society of Clinical Oncology-College of American Pathologists), WHO guidelines, and also based on annotation metrics from OncoMD (a knowledge base of genomic alterations).

Results: The informativity of the panel was 95 percent. *NOTCH 1* was the most frequently mutated gene in 16.1% of patients followed by 12.9% who had ARID1A mutations. *MYD88* and *TP53* mutations were detected in 9.6% of the patient while 6.4% of patients had *CSF3R* mutations. *NOTCH 1 *and *TP 53* are the most frequently reported gene in the middle age group (40-60). Mutation in *MYD88* is reported in every age group. *MYD88* (51%) is the most common mutation in ABC subtypes of DLBCL, followed by *NOTCH 1* (44%) and *SOCS 1 *(33%) according to our findings. *NOTCH 1 *mutations are frequent in ABC and PMBL subtypes. Closer investigation reveals missense mutation is the most frequent mutation observed in the total cohort targeting 68.4% followed by frameshift deletion reported in 26.3%. Six novel variants have been discovered in this study.

Conclusions: This study demonstrates the high yield of information in DLBCL using the NGS Lymphoma panel. Results also highlight the molecular heterogeneity of DLBCL subtypes which indicates the need for further studies to make the results of the NGS more clinically relevant.

## Introduction

Among adult lymphoma, diffuse large B-cell lymphoma (DLBCL) is the commonest with 30-40% of global incidence [1]. DLBCL's molecular heterogeneity understanding has been much advanced due to gene expression profiling (GEP) studies allowing classification as germinal center B-cell-like (GCB), activated B-cell-like (ABC), and primary mediastinal B-cell lymphoma (PMBL) also referred to as cell-of-origin (COO) classification [2,3]. The hallmark of the GCB subtype is the loss of PTEN (a tumor suppressor) and chromosomal translocation t(14;18)(q32;q21) juxtaposing the *BCL2* gene [4,5]. ABC subtype prominently reveals deletion of the INK4/ARF locus, *BCL2* amplification, and 3q27 rearrangements involving *BCL6* [6,7]. PMBL has classical Hodgkin lymphoma (CHL) like molecular hallmarks including *JAK2* amplifications and *SOCS1* deletions [8,9].

The clinical presentation, immunochemotherapy response, and the outcome of these subtypes differ. The ABC subtype has the worst prognosis whereas the GCB subtype has the comparatively best responses [2,7]. The availability of targeted medicines has made the identification of each molecular subtype essential so that each patient can receive the best possible therapy.

Despite recent breakthroughs in bench-to-bedside translation, the GEP-based classification has little influence on clinical practice due to difficulty in applying it for routine diagnosis due to molecular heterogenicity of disease [10-12]. The answer to this has emerged as high-throughput DNA sequencing in form of next-generation sequencing (NGS) allowing insights and detailed genomic characterization of DLBCL by identifying recurrent single nucleotide variants (SNV) prevalent in specific subtypes [13-22]. Despite the diversity, SNVs are particularly significant since their presence in actionable targets is associated with therapy response.

Moreover, notwithstanding pathophysiologic significance, the prediction of resistance to therapy or prognostic implications to immunochemotherapy treatment have not been thoroughly investigated in clinical trials. Additionally, activation-induced cytidine deaminase (AID)-driven somatic hypermutation (SHM) regulating the creation of recurrent SNVs, makes it difficult to distinguish between driver and passenger changes [23].

Hence to identify molecular subgroups and ultimately move towards precision treatment, requires a suitable panel of target genes NGS to uncover and describe the SNVs harbored by DLBCL patients. We devised an NGS lymphoma panel test after reviewing previous studies in DLBCL [13,14,20,23,24]. In the current study, the lymphoma panel was employed to sequence 20 patients diagnosed after a centralized histopathologic review. This study attempted to achieve information using the NGS lymphoma panel that can shed a light on the molecular heterogeneity of DLBCL subtypes.

## Materials and methods

This study included 25 DLBCL patients after a centralized histopathologic review. The quality and quantity of tumor cells were accessed by H&E staining and correlated with histopathology and immunohistochemistry (IHC) status. Patients were grouped as ABC, PMBL, and "other" or unclassified subtypes based on Hans algorithm. Five patients not having complete clinical information and immunohistochemistry data were excluded from this study. The study finally included 20 patients having DLBCL. Institutional Ethics Committee, All India Institute of Medical Sciences (AIIMS), Rishikesh approved (approval no. AIIMS/IEC/20/592) this study, and all participants gave written informed consent.

Pretreatment evaluation included complete physical examination, routine blood testing, and serum chemistry profile, including lactate dehydrogenase (LDH) levels and serum β2-microglobulin. Additionally, computed tomography (CT) scans findings of the neck, chest, abdomen, and pelvis, and bone marrow examination were also noted. The Ann Arbor staging system was used. Immunohistochemically classified the enrolled patients into different subtypes [13].

The lymphoma panel of 133 was designed on targeted sequencing of multiple genes for the coding regions through NGS. The scope of this lymphoma panel included a panel of genes, wherein the prognostic significance of these genes and their mutations were intended to be studied. Previous publications were used to design it.

The cellularity of samples was checked before analysis. Targeted gene capture using a custom capture kit was done for genomic DNA. The libraries were prepared and sequenced using the Illumina platform. The human reference genome (GRCh38.p13/) alignment of obtained sequences was performed using Burrows-Wheeler Aligner (BWA) program [24,25]. Identification of somatic mutations was done using LoFreq (version 2) variant caller [26,27]. For clinical interpretation, only non-synonymous and splice site variants revealed in the coding regions were utilized.

The mutations were annotated using an annotation pipeline (VariMAT). Ensembl Variant Effect Predictor (VEP) program release 99 human gene Model was used for gene annotation of the variants [28,29]. Previously published literature and databases were used for the annotation of clinically relevant mutations. The common variants were filtered for reporting based on the presence in various population databases (1000G, ExAC, EVS, 1000Japanese, dbSNP, UK10K, MedVarDb (in-house database) [30-34]. A custom read-depth-based algorithm was used to determine CNV (Copy Number Variants) from targeted sequencing experiments. Rare CNVs were detected using a comparison of the test data read-depths with the matched reference dataset. Reportable mutations were prioritized and prepared based on AMP-ASCO-CAP (Association for Molecular Pathology-American Society of Clinical Oncology-College of American Pathologists), WHO guidelines [35,36], and also based on annotation metrics from OncoMD (a knowledge base of genomic alterations) [37].

## Results

**Figure 1:**
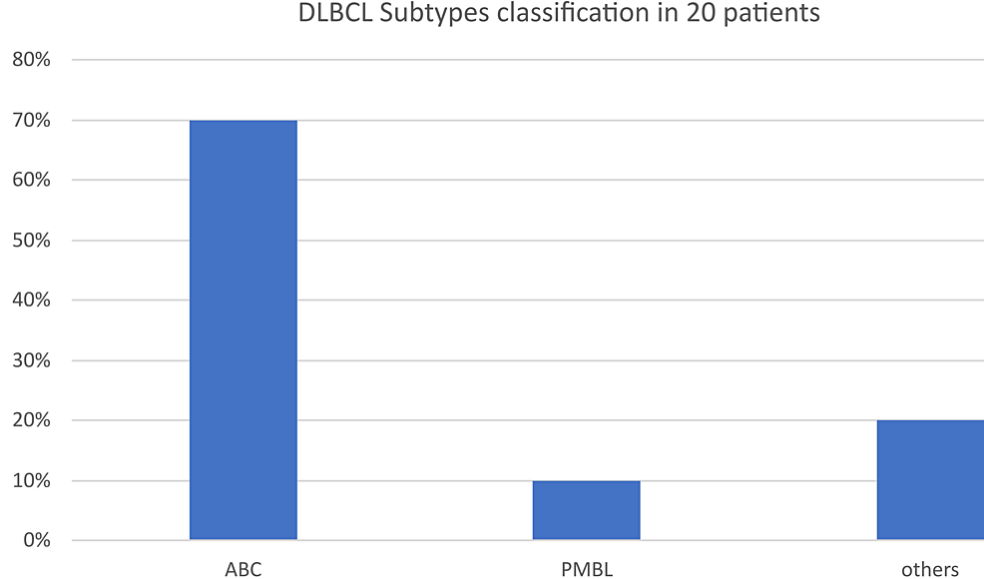
DLBCL subtypes classification in 20 patient DLBCL: Diffuse large B-cell lymphoma

Lymphoma panel was performed on samples of only 20 DLBCL patients with a median age of 56 (range, 15-75) years. ABC accounts for 70% (14/20) of the patients, PMBL accounts for 10% (2/20), and other accounts for 20% (4/20) of the patients as depicted in Figure 1.

According to the lymphoma staging classification system, 40% of the patients included have AnnArbor stage 4. While 50% have stage 3. International Prognostic Index (IPI) scores categorize patients as having Low, Low-intermediate, and High-intermediate risk. In our cohort 52% of patients are at high intermediate risk, 33% are at low intermediate risk and only 15% of the patient are having the low-risk category. Total cohort 88% of the patients exhibited Clinical B symptoms.

**Figure 2:**
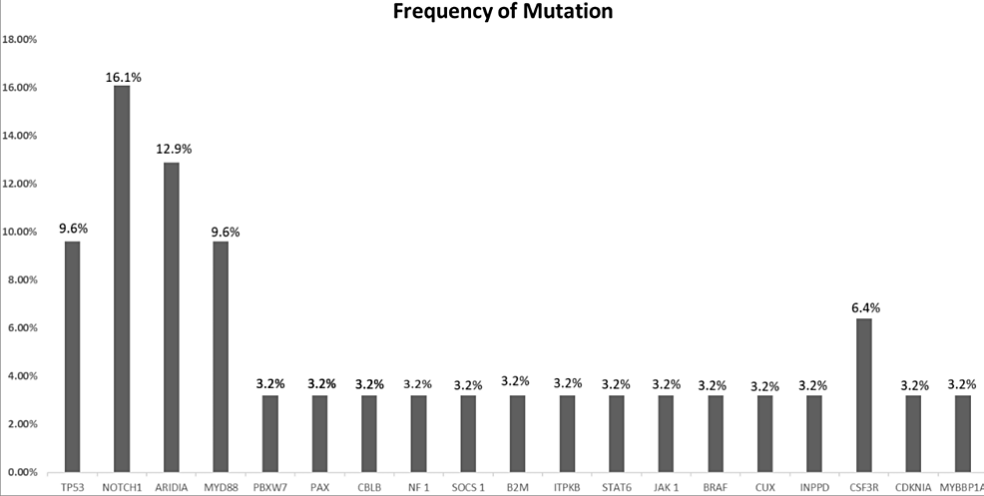
Mutation frequencies in the 20 patients.

The panel informativity was 95 percent. Nineteen out of 20 included subjects had at least one mutation. Variants per gene were found to be ranging from one to eight. Somatic mutational landscape examination after sequencing of all the genes yielded a result that among all the mutant genes 16.1% of patients had *NOTCH 1 *mutations followed by 12.9% had *ARID1A* mutations. *MYD88* and *TP53* genes had a 9.6% mutation rate and *CSF3R* had a 6.4%. The rest of the genes like *FBXW7*, *PAX5*, *NF1*, *SOCS1*, *BRAF*, *INPPD*, *CUX1*, *JAK2*, *CBL*, and *CDKN1A* accounts for 3.2% of all mutations. Gene mutation frequencies in the various subtype are illustrated in Figure 2.

**Figure 3:**
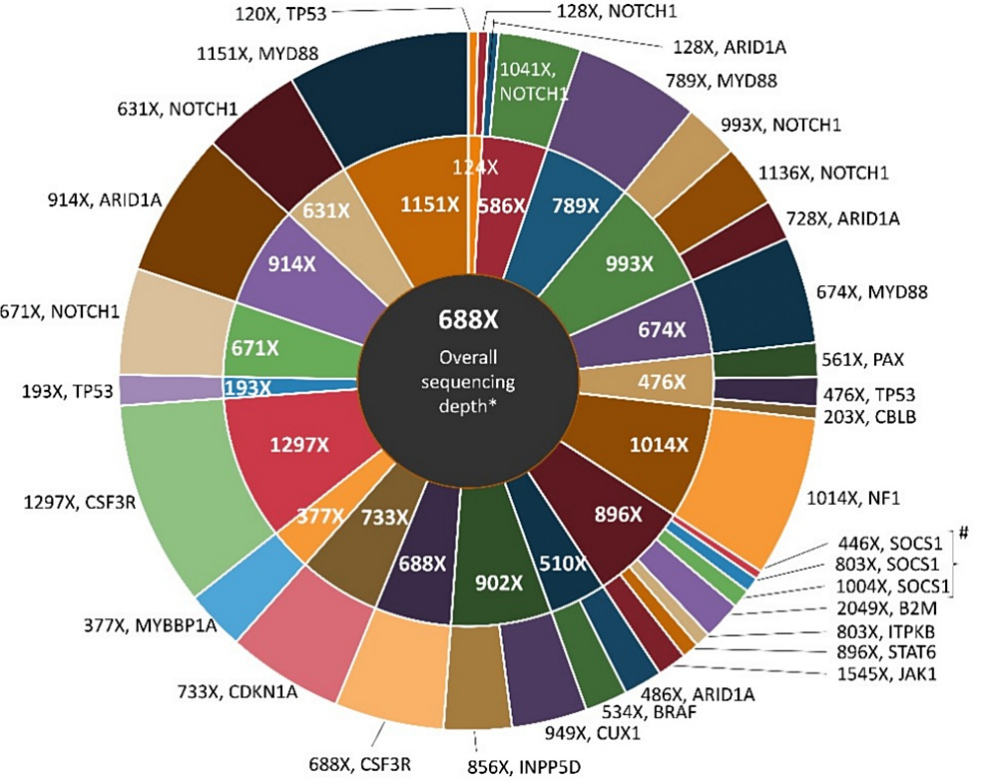
Overall Median Depth of Mutation #one gene with different CDS (coding sequence) variants and amino acid change

The median overall sequencing depth for the lymphoma panel was 688X (120X-2049X), for individual genes sequencing depth is different as presented in Figure 3. The inner circle denotes the average depth for each patient and the outer ring shows the depth for each mutation for that particular patient.

**Figure 4:**
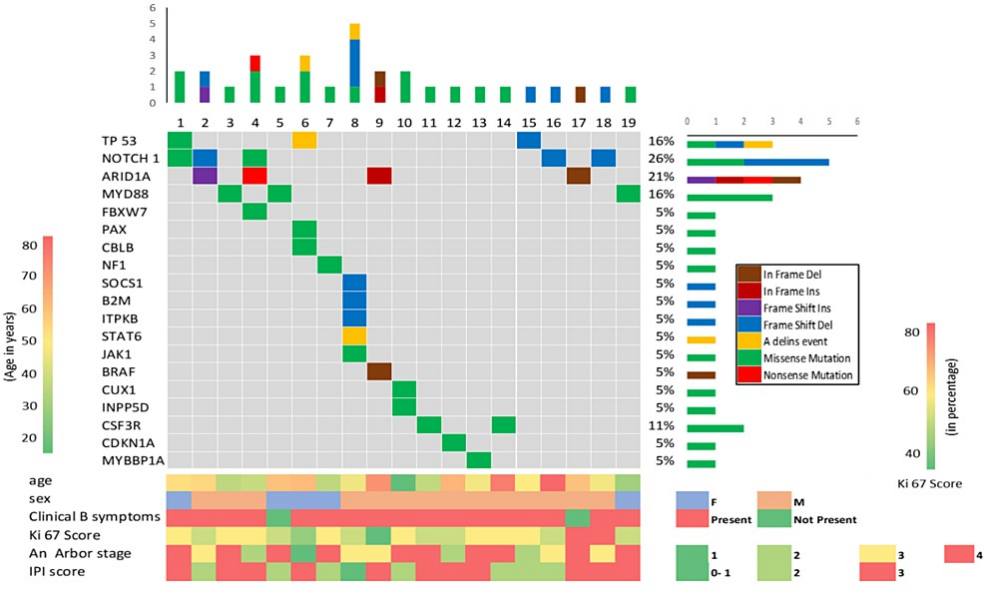
- Somatic Mutation landscape In this landscape, a horizontal lane depicts a single gene and the vertical lines represent different mutations. One patient with no mutation is excluded in this figure. Different colors indicate mutation type. Schematic depiction of age, sex, clinical B symptoms, Ki 67 score, Ann arbor staging, and IPI scores with patients grouped by age ( 20-30 green, 30-40 light green, 40-50 yellow, 50-60 orange, and above 60 red color) and Clinical B symptoms is also shown (Red is Present and green is not present), Percentage of Ki 67 shown as (above 70 years old shown in red, between 50 to 60 year old shown in yellow and below 50 are shown in green), AnnArbor stage also indicated for each patient ( Dark green 0-1, light green 2, yellow 3 and Red 4) and IPI score is indicated as 0-1 green color, 2 light green color, and 3 red colors). IPI: International Prognostic Index

Each finding in the lymphoma panel was correlated with clinical features like age, stage, IPI score, etc. The included cohort was divided into age groups of (n=2) 20 to 40 years, (n=9) 40 to 60 years, and those belonging to the (n=9) 60-80 age group. Only three mutations were observed in the younger age group (*MYD88*, *CUX1*, and *INPP5D *mutations). Genetic analysis of one patient of this age group revealed no mutation. Whereas *NOTCH 1* and *TP 53* are the most frequently reported gene in the middle age group (40-60). *PAX*, *CBLB*, *BRAF,* and *CDKN1A* frequency are reported only in the 60-80 years of age group. Mutation in *MYD88* is reported in every age group. Of 20 patients, 15 patients are male and the most common mutations in male is in *NOTCH 1* and *ARID1A* (16% each), followed by *SOCS1* (12%) and *CSF3R* (8%) whereas in females commonest mutations are *MYD88* and *TP53* (25% each). *PAX*, *CBLB,* and *NF1* mutations are found only in the females included in the present study. According to IPI Score, patients having score 1 most commonly have a mutation in *SOCS1* (43%). Those having a score 2 have *NOTCH 1* (22%) and enrolled patients with a score 3, both *NOTCH 1* and *ARID1A* (18%). As per Ann Arbor, most common mutations in stage 4 patients were in the *MYD88* gene followed by *CSF3R* (17%), whereas in stage 3 patients *SOCS 1* and *NOTCH 1 *(23%) followed by *ARID1A* (15%) were commonly mutated and in stage 2 and stage 1 patients had mutated *TP53 *gene. According to the Ki67 score, in the 60-80% score group, the most common mutations are in the genes of *NOTCH 1* (21%) followed by *ARID1A* and *TP53*. For those having scores of 40-60, the commonest mutations were found in *SOCS* (18%) followed by *MYD88* and *NOTCH 1* (12%). Overall *TP53* mutations were found in all stages. These are illustrated in Figure 4.

All of the gene alterations in our population were categorized into three subtypes of DLBCL. ABC, PMBL, and Other. *MYD88* (51%) is the most common mutation in ABC subtypes of DLBCL, followed by *NOTCH 1* (44%) and *SOCS 1 *(33%) according to our findings. *ARID1A*, *CBLB*, *CUX1*, *JAK 1*, *NF 1*, *NOTCH 1*, and *STAT 6* are the most commonly reported mutations in PMBCL subtypes. *TP 53* is the most prevalent mutation in other subtypes, while *NOTCH 1* is the most common mutation in DLBCL patients with the ABC and PMBL subtypes.

Again, upon closer investigation missense mutation is the most frequent mutation observed in the total cohort targeting 68.4% of the total cohort followed by Frameshift deletion reported in 26.3% of the total cohort. *NOTCH 1* involves many Frameshift deletion mutations while the *ARIDIA*-only gene includes four different types of mutations (frameshift insertion, in-frame insertion, nonsense mutation, and in-frame deletion mutation). Notably, *MYD88* and *TP 53* include many missense mutations in 32% of the samples followed by some frameshift deletions reported in *TP 53* mutation reported in this cohort.

Novel mutations

The lymphoma panel identified many novel mutations which could potentially serve as means to stratify patients and possibly align with treatment options. These were mostly loss function mutations and these variants have not been reported in ExAC and 1000 genomes database.

In one patient having ABC subtype, a frameshift deletion (chr9:g.136496514del; c.7225del) resulted in prematurely truncated protein (downstream to codon 2409 at 13 amino acid, p.Gln2409SerfsTer13) was spotted in the *NOTCH 1* gene. Another variant detected in the sample of the same patient was a nonsense mutation (chr1:g.26731170C>T; c.1369C>T) in the *ARID1A* gene resulting in the introduction of a stop codon and truncation of the protein at codon 457 (p.Gln457Ter). A frameshift deletion (chr17:g.7675181_7675197del; c.415_431del) leading to prematurely truncated protein (at codon 139, only 4 amino acids downstream of the said codon, p.Lys139AlafsTer4) was detected in the *TP53* gene of this same patient.

A sample of another patient having PMBL revealed frameshift deletion in the *SOCS1* gene (chr16:g.11255131del; c.348del) resulting in termination of the protein (2 amino acids downstream to codon 116, p.Ser116ArgfsTer2). Another frameshift deletion in the *SOCS1* gene (chr16:g.11255444_11255457del; c.22_35del) resulting in subsequent termination of the protein (at codon 8, 104 amino acids downstream, p.Ala8SerfsTer104) was detected in same patient. A frameshift deletion (chr16:g.11255019_11255029del; c.450_460del) resulting in termination of the protein, (downstream to codon 151, p.Leu151ArgfsTer98) was detected in the *SOCS1* gene of same patient.

## Discussion

A lymphoma panel was performed on samples of only 20 DLBCL patients with a median age of 56 (range, 15-75) years. ABC accounts for 70% (14/20) of the patients, PMBL accounts for 10% (2/20), and other accounts for 20% (4/20) of the patients as depicted in similar results previously reported indicating molecular heterogeneity of DLBCL with a preponderance of ABC subtypes [38].

The lymphoma panel was designed for targeted sequencing of multiple genes for the coding regions through NGS. Our study included 133 genes and there are studies with multiple genes [38,39]. The informativity of our lymphoma panel was 95 percent similar to previous studies [38]. One patient sample did not yield genetic variation. The number of variants per gene was found to be ranging from one to eight.

On examination of the somatic mutational landscape, we found out that among all the mutant genes 16.1% of patients had *NOTCH 1* mutations followed by 12.9% *ARID1A* mutations. Mutation analysis in many studies has delineated that *NOTCH 1* mutations are one of the distinctive drivers of DLBCL [38,40]. Schmitz et al. developed a molecular classification based on 574 cases of DLBCL using various methods to identify genetic subtypes [41]. The four subtypes given by them include EZB, BN2, MCD and N1. The genetic hallmarks of BN2 subtypes include BCL6 fusions and variations of the pathway of NOTCH as found in our study. *ARID1A* mutations are common in ovarian clear cell carcinoma. Also detected in the 2-17 % range in myeloblastoma, neuroblastoma, Burkitt lymphoma, and Waldenstrӧm macroglobulinemia. Along with other genes (*HIST1H1C/D/E*, and *SMARCA4*), the *ARD1A* gene, one of the regulators of higher-order chromatin structure, has been reported to be mutated in ⩾5% of DLBCL or follicular lymphoma [42]. In our study, a much higher yield of 12.9% was seen, with *ARID1A* in all subtypes. This difference needs further studies since this has not been documented in Indian DLBCL patients previously, this finding is significant and further studies are required to give insight.

*MYD88* and *TP53* genes had a 9.6% mutation rate and *CSF3R* had a 6.4%. *MYD88* (40%) is the most common mutation in ABC subtypes of DLBCL gene alterations, according to our findings. Our findings have been mirrored by previous reports of *MYD88* mutation being reported in 30-40% of ABC type DLBCL [43], although lower frequencies have been reported by others [43,44]. *MYD88* has been selected as an essential mutation in the ABC [45,46]. The rate of TP53 variants present in our study was less than in previously reported publications [38]. Our study has 12% of samples showing *SOCS1 *mutations while the previous study has reported higher (55.5%) PMBL patients [38].

In the present study, the cohort was divided into three age groups. *PAX*, *CBLB*, *BRAF*, and *CDKN1A* frequency are reported only in the 60-80 years of age group. *NOTCH 1 *and *TP 53* are the most frequently reported gene in the middle age group (40-60). We observed only three mutations in the younger age group (*MYD88*, *CUX1*, and *INPP5D*). Mutation in *MYD88* is reported in every age group. in a previous study, authors found a correlation between age with *CD79B*, *KMT2D*, and *MYD88* mutations [38].

According to IPI Score in our study, in Score 1 most common mutation is found in *SOCS1* (43%) and score 2 *NOTCH1* (22%) and score 3, both *NOTCH1* and *ARID1A* (18% ) followed by *MYD88* and *TP53* (12% each). The comparison of four independent DLBCL studies done by Mellert et al. suggests that *SOCS1* mutations may be used to forecast an extremely favorable overall survival rate of early-onset DLBCL patients (aged <60 years) [47]. Similarly, it is known that *TP53* alterations are more frequently associated with a lack of response to first-line chemotherapy [48] while *MYD88* mutations signal adverse prognostic impact [49]. Both these findings are mirrored in our study with lower score patents having higher SOCS1 mutation and those having an IPI score of 3 having a high percentage of *TP53* and *MYD88* mutations.

The investigation also yielded missense mutation to be the most frequent mutation observed in the total cohort targeting 68.4% of the total cohort followed by frameshift deletion reported in 26.3% of the total cohort. *NOTCH 1* involves many frameshift deletion mutations while the *ARIDIA*-only gene includes four different types of mutations (frameshift insertion, in-frame insertion, nonsense mutation, and frame deletion mutation). Notably, *MYD88* and *TP 53* include many missense mutations in 32% of the samples followed by some frameshift deletions reported in the *TP53* mutation reported in this cohort. A study by Fan et al. concluded that out of the total, somatic mutations detected in DLBCL, foremost were missense type, followed by silent, splice-site, nonsense, and indels. Of the indels, maximum caused reading frameshifts [50].

In our present study, the lymphoma panel identified many novel mutations. These included a frameshift deletion in the *NOTCH1* gene, a nonsense mutation in the *ARID1A* gene, frameshift deletions in the *SOCS1* gene, and frameshift deletion in the *TP53* gene. These novel mutations can potentially be used for the stratification of patients in the future according to linking them to treatment options in coming studies.

Study limitations

The study's main limitations include its small sample size and lack of follow-up.

## Conclusions

The importance of the information provided by the NGS lymphoma panel in patients with DLBCL is demonstrated in this study. Findings highlight the subtype molecular heterogeneity and identify novel mutations. This study's results point toward the importance of NGS, giving information about well-documented genes as well as novel mutations which can be potential targets in the future. The study adds to already existing knowledge significantly. Lymphoma panel has identified many novel mutations which could potentially have clinical applications in terms of stratification of patients according to treatment options. The major challenge which remains is pinpointing a clinical relevance. There clear need for an all-inclusive resource annotating associations of revealed genetic alterations with various pathophysiological pathways, possible drug targets, and their effect on therapy response. Further studies with NGS in DLBCL will make it possible to explore genetic variants and annotate and associate them with the phenotype for proper utilization in clinical settings.
